# *Chlamydia trachomatis* induces lncRNA MIAT upregulation to regulate mitochondria‐mediated host cell apoptosis and chlamydial development

**DOI:** 10.1111/jcmm.17069

**Published:** 2021-12-03

**Authors:** Fangzhen Luo, Yating Wen, Lanhua Zhao, Shengmei Su, Yuqi Zhao, Wenbo Lei, Zhongyu Li

**Affiliations:** ^1^ Institute of Pathogenic Biology Hengyang Medical College Hunan Provincial Key Laboratory for Special Pathogens Prevention and Control University of South China Hengyang China; ^2^ Hunan Polytechnic of Environment and Biology Hengyang China

**Keywords:** apoptosis, *Chlamydia trachomatis*, lncRNA MIAT, mitochondrial pathway, persistent infection

## Abstract

*Chlamydia trachomatis* persistent infection is the leading cause of male prostatitis and female genital tract diseases. Inhibition of host cell apoptosis is the key to maintaining *Chlamydia* survival in vivo, and long noncoding RNAs (lncRNAs) play important roles in its developmental cycle and pathogenesis. However, it is not clear how lncRNAs regulate persistent *Chlamydia* infection. Here, using a microarray method, we identified 1718 lncRNAs and 1741 mRNAs differentially expressed in IFN‐γ‐induced persistent *C*. *trachomatis* infection. Subsequently, 10 upregulated and 5 downregulated differentially expressed lncRNAs were verified by qRT–PCR to confirm the reliability of the chip data. The GO and KEGG analyses revealed that differentially regulated transcripts were predominantly involved in various signalling pathways related to host immunity and apoptosis response. Targeted silencing of three lncRNAs (MIAT, ZEB1‐AS1 and IRF1) resulted in increased apoptosis rates. Furthermore, interference with lncRNA MIAT caused not only an obvious downregulation of the Bcl‐2/Bax ratio but also a marked release of cytochrome c, resulting in a significantly elevated level of caspase‐3 activation. Meanwhile, MIAT was involved in the regulation of chlamydial development during the persistent infection. Collectively, these observations shed light on the enormous complex lncRNA regulatory networks involved in mitochondria‐mediated host cell apoptosis and the growth and development of *C*. *trachomatis*.

## 1 INTRODUCTION

*Chlamydia trachomatis* is a strict intracellular parasitic gram‐negative prokaryotic microbe. Persistent *C*. *trachomatis* infection can cause urethritis, pelvic inflammatory disease, ectopic pregnancy, oocyte sterility and other diseases.^1^ Although *C*. *trachomatis* infection is currently treated effectively with antibiotics,^2^, ^3^ with the absence of symptoms often leading to delays in diagnosis and treatment, infection rates continue to rise worldwide.^4^

One of the most successful studies was the IFN‐γ‐induced persistent *C*. *trachomatis* infection model,^5^ and it has been shown that under the treatment of IFN‐γ, *C*. *trachomatis* forms an atypical infective state in host cells. Although inclusion bodies still exist, they have no infective capacity.^6^ Under the electron microscope, a large number of abnormally enlarged and loose reticulate bodies (RBs) were found in the inclusions, which we called abnormal bodies (ABs). Mature elementary bodies (EBs) are not seen or are rarely seen.^7^

Apoptosis is an important defence mechanism against pathogens.^8^ The regulation of *C*. *trachomatis* infection on apoptosis is quite complex. This may be due to the combination of host immune clearance and *Chlamydia* immune escape. At present, it is believed that *C*. *trachomatis* has a significant time effect on the regulation of cell apoptosis, which can inhibit the apoptosis of host cells in the early stage of infection and induce the apoptosis of host cells in the late stage of infection.^9^, ^10^ Therefore, the molecular mechanism of *C*. *trachomatis* antiapoptotic activity may be one of the causes of persistent host infection.

Studies have shown that intracellular pathogens can regulate cell signalling pathways and achieve immune escape by regulating the expression profiles of host lncRNAs,^11^ such as *Rickettsial*,^12^ *Mycobacterium tuberculosis*,^13^ *Helicobacter pylori* ^14^ and *Listeria monocytogenes*.^15^ *C*. *trachomatis*, as an intracellular pathogen, causes physiological changes in the host similar to those of the virus and changes the expression profile of the host mRNA, thus promoting its infection. However, lncRNAs can affect the expression of host mRNA through direct binding of mRNA or competitive binding of miRNA,^16^ which suggests that *C*. *trachomatis* may construct a complex intracellular regulatory network by changing the expression profile of host lncRNAs and thus fine‐regulated cellular gene expression. To influence the process of persistent infection and *C*. *trachomatis* intracellular survival, the interaction mechanism remains to be further studied. The role of lncRNAs in bacterial infection has aroused widespread concern,^17^, ^18^ and the molecular mechanism of *C*. *trachomatis* pathogenesis has not been fully elucidated; therefore, the study of host lncRNAs in *C*. *trachomatis* infection may provide new ideas for the treatment of *Chlamydia* infection.

In the present study, transcriptomic profiles of lncRNAs and mRNAs from persistent *C*. *trachomatis*‐infected HeLa cells were performed using a microarray platform. Our results identified changes in the expression patterns of lncRNAs and mRNAs between IFN‐γ‐induced *C*. *trachomatis* infection and IFN‐γ control samples. The results demonstrated that the upregulation of lncRNA MIAT‐regulated mitochondria‐mediated host cell apoptosis and chlamydial development during persistent infection. This study may provide new insights into the pathogenic mechanisms associated with persistent *C*. *trachomatis* infection.

## 2 MATERIALS AND METHODS

### 2.1 *C. trachomatis* and cell culture

HeLa 229 cells were cultured and maintained in DMEM (Dulbecco's modified Eagle's medium; Gibco) containing 10% (v/v) foetal bovine serum (FBS; Gibco) at 37°C in an incubator with 5% CO_2_. The *C*. *trachomatis* E strain used in this study was cultured as described in the previous research.^19^ The standard strain of *C*. *trachomatis* serovar E was obtained from ATCC (ATCC# VR‐348B, BOUR, GenBank# JX559522).

### 2.2 Persistent *C. trachomatis* infection

The cultured HeLa cells were inoculated in 6‐ or 24‐well plates and cultured at 37°C with 5% CO_2_ overnight. Then, 1 ml of DMEM was added to each 6‐well plate (24‐well plate, 200 μl), containing a final concentration of 30 μg/ml DEAE‐dextran, and the plate was incubated at 37°C for 15 min. The *C*. *trachomatis* E strain was diluted to an appropriate concentration with DMEM and added to the cell culture plate. Then, the strain was placed in an incubator at 37°C for 30 min and centrifuged at 1000 rpm for 45 min to assist infection, whereafter the infection solution was discarded. Finally, DMEM containing 75 U/ml IFN‐γ and 10% FBS was added to each well and cultured at 37°C with 5% CO_2_.

### 2.3 RNA extraction and lncRNA microarray

Total RNA was isolated from IFN‐γ‐induced *C*. *trachomatis* infection and IFN‐γ control samples using TRIzol reagent (Invitrogen). We employed Arraystar Human LncRNA Microarray V5.0, which was designed for the global profiling of human lncRNAs and protein‐coding transcripts. Approximately 39,317 lncRNAs and 21,174 coding transcripts could be detected by third‐generation lncRNA microarray. The protocol was as follows: First, mRNA was purified from total RNA after the removal of rRNA (mRNA‐ONLY™ Eukaryotic mRNA Isolation Kit, Epicentre). Second, each sample was amplified and transcribed into a fluorescent cRNA using a random primer method. Third, the labelled cRNAs were purified using an RNeasy Mini Kit (Qiagen), and the concentration and activity were detected by a NanoDrop ND‐1000. Fourth, chip hybridization was performed. Fifth, the hybrid chip was washed, fixed and scanned (Agilent DNA Microarray Scanner (Part Number G2505C)). Differentially expressed lncRNAs and coding transcripts with statistical significance between the two groups were identified through *p*‐value and fold change filtering.

### 2.4 Quantitative real‐time PCR

The total RNA of each sample was measured by a nucleic acid‐protein analyser and then reverse‐transcribed to cDNA by a reverse‐transcription kit (Tiangen) according to the manufacturer's instructions. qRT‐PCR was performed using SYBR Green premix (Tiangen) in a LightCycler 96 apparatus (Roche, Basel, Switzerland). Amplification was performed for 600 s at 95°C, followed by 40 cycles of 95°C for 15 s and 60°C for 30 s. *18S rRNA* acted as an internal control. Three parallel replicates were performed for each sample. The relative expression levels of the lncRNAs LINC01128, MIAT, CYTOR, ZEB1‐AS1, FRMD6‐AS2, NNT‐AS1, LINC00240, EBLN3P, PANDAR, IRF1, LINC00466, SEMA3B‐AS1, KRT7‐AS, HCG18 and LINC00163 were normalized to that of *18S rRNA* within each sample using the 2^−ΔΔCt^ method. Primers designed for validation are shown in Table S1.

### 2.5 Bioinformatics analysis

To display the differential expression of lncRNAs and mRNAs more intuitively, MeV V4.9.0 software was used in this study to cluster the data and obtain the heatmap. Then, Gene Ontology (GO) and Kyoto Encyclopaedia of Genes and Genomes (KEGG) analyses of chip data were performed using the R language package to evaluate the functions of differentially expressed genes. The lncRNA‐miRNA‐mRNA interaction network was constructed using the RNA‐RNA database (http://starbase.sysu.edu.cn/index.php) and visualized using Cytoscape 3.6.1 (https://cytoscape.org/).

### 2.6 RNA interference

Small interfering RNAs (siRNAs) corresponding to the sequences of MIAT, ZEB1‐AS1 and IRF1, which were used to inhibit endogenous expression of the above three lncRNAs, and the negative control (NC) siRNAs, which exhibited no downregulation of any HeLa cell genes, were synthesized by Ribio. Transfection was performed with Lipofectamine 3000 (Invitrogen). Cells were transfected with 50 nm of each siRNA.

### 2.7 Immunofluorescence analysis

The cells were fixed with 4% paraformaldehyde for 30 min, and then 0.1% Triton X‐100 was added and allowed to permeate for 10 min. DMEM containing 10% FBS was used for blocking for 1 h. After washing with PBS twice, primary antibodies (rabbit anti‐*C*. *trachomatis*, rabbit anti‐cytochrome c) were diluted with DMEM at a ratio of 1:500, mixed and incubated in a 37°C incubator for 1 h. Subsequently, the Cy2‐labelled sheep anti‐rabbit fluorescent secondary antibody (1:200) and the nuclear dye Hoechst 33258 (1:1000) were diluted in DMEM to avoid light and incubated for 1h at 37°C. Finally, the inclusions were observed and photographed by an inverted fluorescence microscope.

### 2.8 Mitochondrial membrane potential (MMP) analysis

MMP was observed in HeLa 229 cells stained with JC‐1 (Beyotime) by fluorescence microscopy. One millilitre of cell culture solution and 1 ml of JC‐1 staining solution were added to the six‐well plate and incubated in the cell incubator at 37°C for 20 min. After incubation, the cells were washed with JC‐1 staining buffer twice. Cell culture solution was added and observed under a fluorescence microscope. Accordingly, MMP was assessed by the ratio of red/green fluorescence intensity.

### 2.9 Flow cytometry analysis

Apoptotic cells were detected with YF^®^647A‐Annexin V and propidium iodide (PI) apoptosis kits (US Everbright). Briefly, cells were trypsinized and harvested after appropriate treatment. Then, harvested cells were incubated with YF647A‐Annexin V and PI for 15 min in the dark according to the manufacturer's instructions. The stained cells were determined by flow cytometry (FACSCalibur, BD, USA).

### 2.10 Western blot analysis

Total protein samples were collected for 24 h after infection, and the protein concentration was determined by the BCA method. Then, SDS–PAGE was carried out. According to the molecular weight of the target protein, the transfer time was set to transfer the protein to the PVDF membrane. After the PVDF membrane was blocked with 5% non‐fat milk, the primary antibody was incubated overnight, and then the secondary antibody was incubated after the membrane was washed. Finally, the results were visualized using an enhanced chemiluminescence western blot system G: BoxChemi XXX9 (Syngene, Cambridge, UK). The densities of the protein bands were analysed by Quantity One (Bio‐Rad, USA).

### 2.11 Statistical analysis

All data were presented as $\bar{X}$ ± SD. Data were analysed and visualized with SPSS 18.0 and GraphPad Prism 5.0. The statistical significance of differences between different groups was analysed with a two‐tailed Student's t‐test. Statistical significance was set at *p* < 0.05.

## 3 RESULTS

### 3.1 Validation of persistent *C. trachomatis* infection in HeLa cells

**FIGURE 1:**
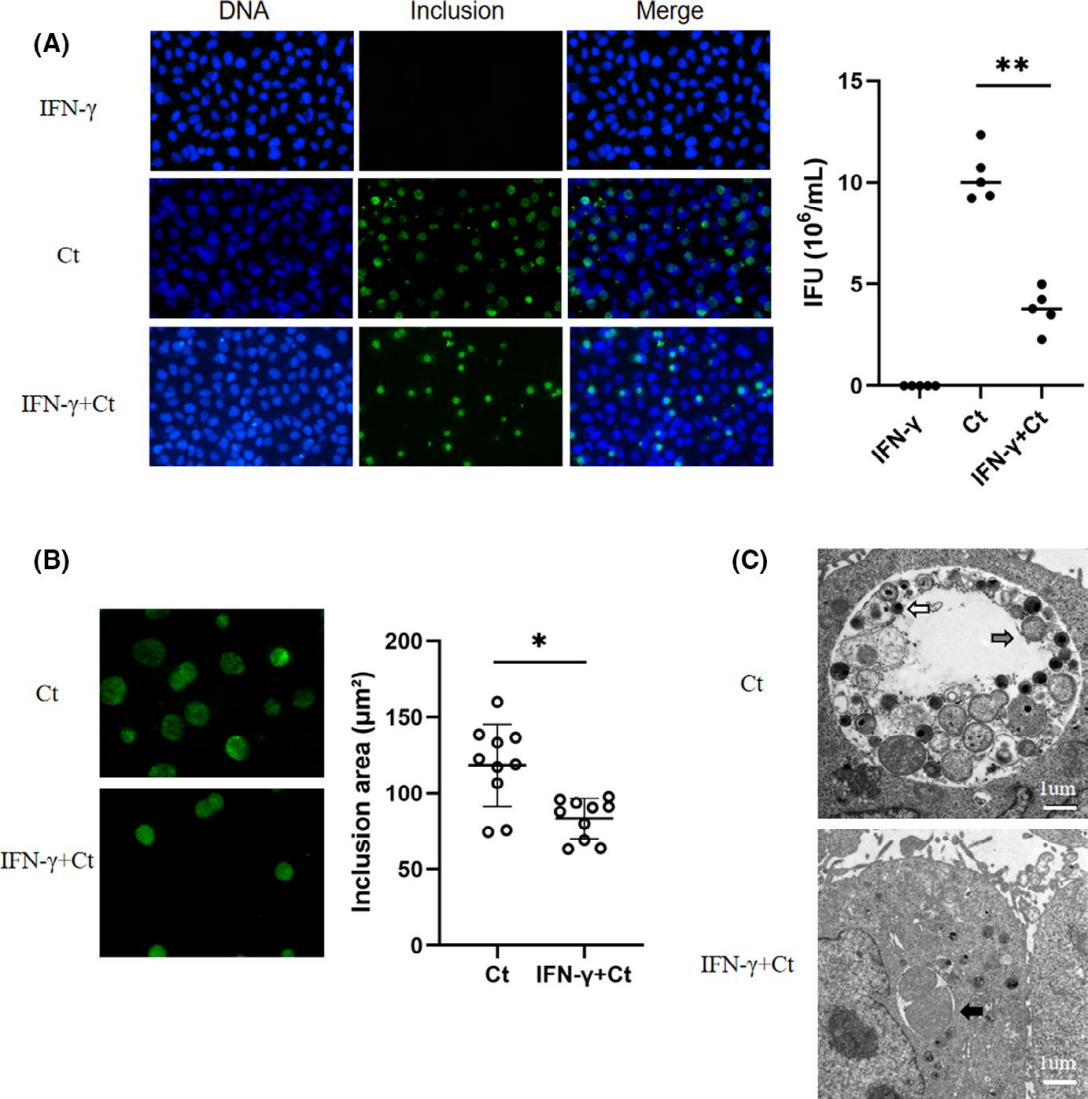
Characterization of persistent *C*. *trachomatis* infection. For the acute infection group, HeLa cells were infected in a 24‐well plate with identical titres (inclusion forming units, IFUs) for 2 h. The persistent infection group was cultured with IFN‐γ (75 U/ml) after infection under the same conditions, whereas the control group was treated with IFN‐γ (75 U/ml) without infection. Samples were observed by microscope simultaneously. (A) Effect of IFN‐γ on the *C*. *trachomatis* infection rate. The number of inclusions expressed as IFUs. DNA (blue) was stained with Hoechst 33258, and inclusions (green) were stained with Cy2. **p < 0.01. (B) Morphological changes in *C*. *trachomatis* inclusions in persistent infection (1000×). The statistical graph shows the areas of the inclusions. The areas were counted by ImageJ. *p < 0.05. (C) The inclusions in cells were mixed and detected by TEM. EBs are indicated by white arrows, RBs are indicated by grey arrows, and ABs are indicated by black arrows; scale bar = 1 µm

In our analysis, we established a persistent *C*. *trachomatis*‐infected HeLa cell model induced by IFN‐γ, and immunofluorescence and transmission electron microscopy (TEM) analyses were used to confirm the persistent infection status in the cell model.^20^ Compared with the IFN‐γ control group, there was no significant change in the cell status of the *C*. *trachomatis* infection group. However, we observed a 3‐fold reduction in the number of inclusions in the persistent infection compared to the acute infection (*p *< 0.01) (Figure 1A), and the inclusion volume was significantly reduced (*p *< 0.05) (Figure 1B). In addition, compared with acute infection, AB with a loose structure and abnormal volume was found in the inclusions of persistent infection, whilst EB and RB were rare (Figure 1C). In summary, our research successfully validated IFN‐γ‐induced persistent *C*. *trachomatis* infection in HeLa cells.

### 3.2 Microarray data analysis of differentially expressed lncRNAs and mRNAs

**FIGURE 2:**
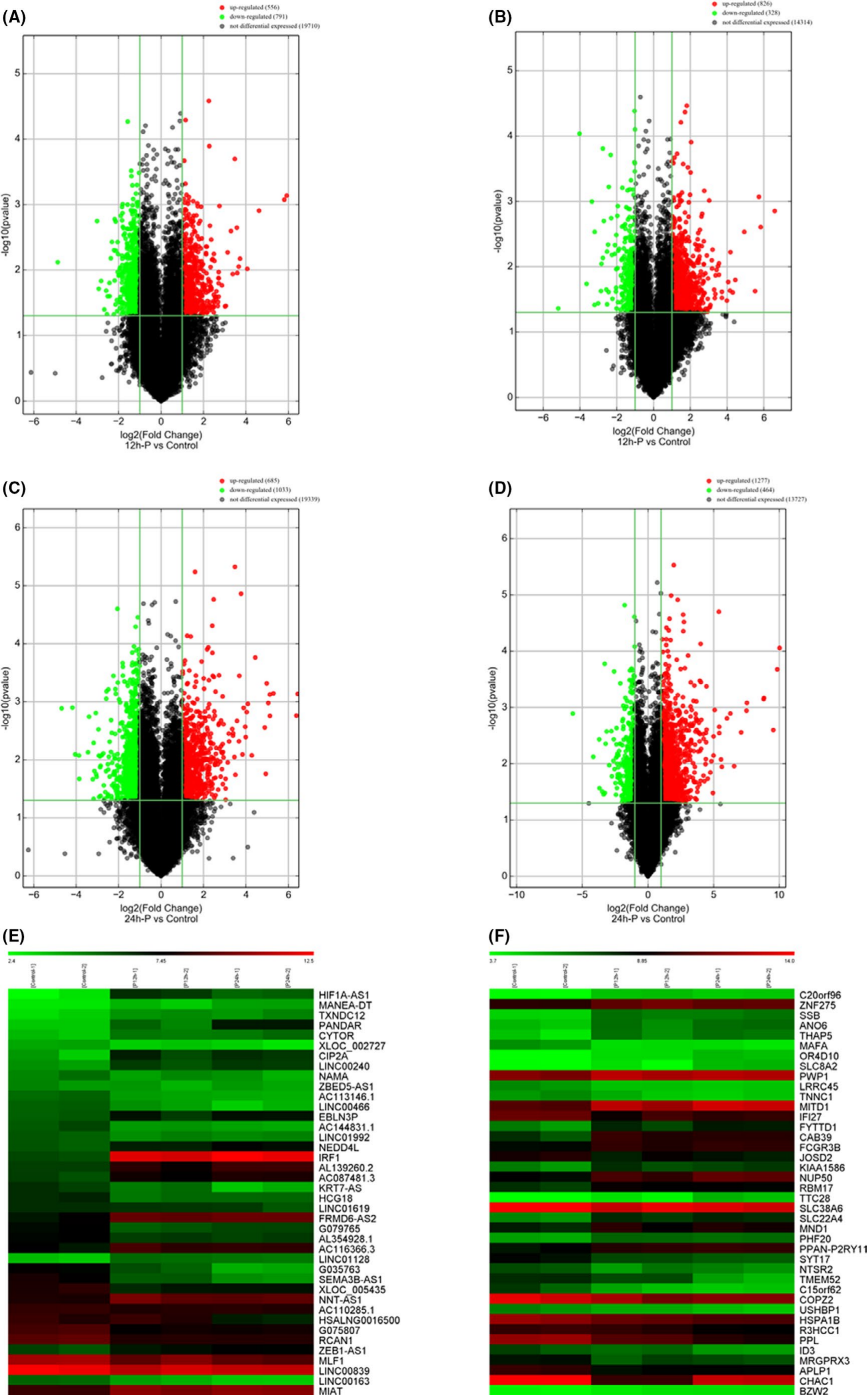
Expression profiles of mRNAs and lncRNAs in the persistent infection and IFN‐γ control groups. (A, C) Volcano plot of lncRNAs at 12 hpi (A) and 24 hpi (C) between the persistent infection and IFN‐γ control groups. (B, D) Volcano plot of mRNAs at 12 hpi (B) and 24 hpi (D) between the persistent infection and IFN‐γ control groups. Upregulated (red), downregulated (green), no expression difference (black). (E, F) Hierarchical clustering of differentially expressed lncRNAs (E) and mRNAs (F) between the 12 hpi, 24 hpi and IFN‐γ control groups. Red indicates relatively higher expression, whereas green indicates relatively lower expression

To identify changes in the expression levels of lncRNAs and mRNAs in an in vitro model of persistent *C*. *trachomatis* infection, total RNA was extracted from IFN‐γ‐induced persistent *C*. *trachomatis*‐infected or IFN‐γ control groups at 12 hpi and 24 hpi. We employed a human lncRNA microarray V5.0 (8×60K, Arraystar, Rockville, MD, USA) containing ~39,317 lncRNAs and 21,174 coding transcripts to screen the differentially expressed lncRNAs and mRNAs. Quantile standardization and subsequent data processing of the original data were performed using GeneSpring GX V12.1 software. LncRNAs or mRNAs with statistically significant differences between the two groups were screened by *P*‐value <0.05 and fold change (FC) of expression ≥2.0. Volcano plots (Figure 2A, B, C, D) were used to show variations in lncRNA and mRNA expression between the persistent infection and IFN‐γ control groups. Compared with the IFN‐γ control group, 1,347 differentially expressed lncRNAs were identified at 12 hpi; amongst them, 556 lncRNAs were upregulated and 791 lncRNAs were downregulated, and 1154 differentially expressed mRNAs were identified; 826 mRNAs were upregulated and 328 mRNAs were downregulated. At 24 hpi, 1718 differentially expressed lncRNAs were identified, amongst which 685 were upregulated and 1033 were downregulated, and 1741 mRNAs were differentially expressed, amongst which 1277 were increased and 464 were decreased. According to the above differentially expressed data, we found that the differentially expressed genes were enlarged at 24 h compared with 12 h, and the differentially expressed values of most genes were increased. A heatmap was used to show 40 differentially expressed lncRNAs and mRNAs in detail (Figure 2E,F), which also provided the basis for subsequent screening and identification. Table S2 and Table S3 summarize the top 20 differentially expressed lncRNAs and mRNAs.

### 3.3 GO and KEGG analyses of differentially expressed mRNAs

**FIGURE 3:**
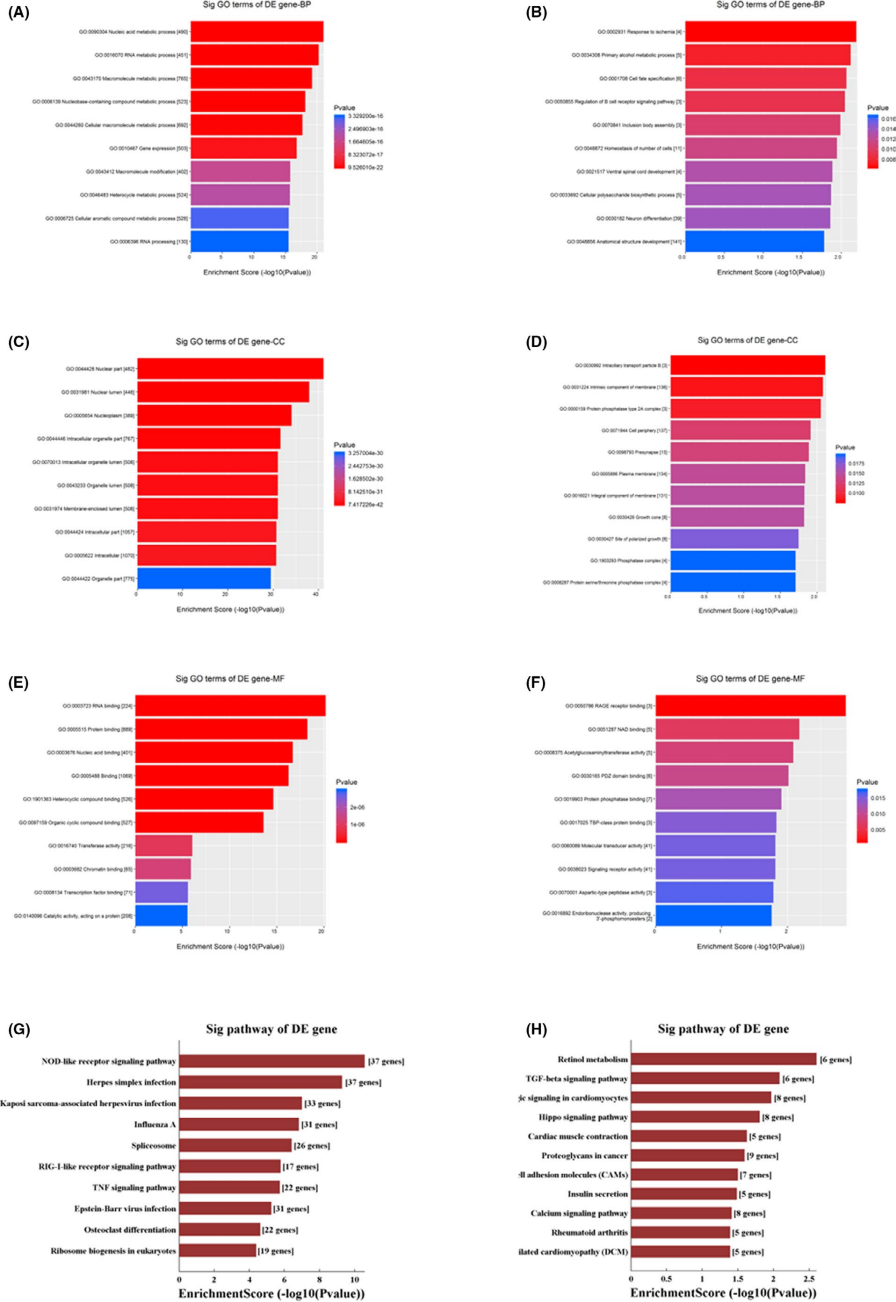
GO categories and KEGG analysis of differentially expressed genes in persistent infection. (A, C, E) The GO categories of upregulated genes at 24 hpi between the persistent infection and IFN‐γ control groups. (B, D, F) The GO categories of downregulated genes at 24 hpi between the persistent infection and IFN‐γ control groups. (G) KEGG analysis of upregulated genes at 24 hpi between the persistent infection and IFN‐γ control groups. (H) KEGG analysis of downregulated genes at 24 hpi between the persistent infection and IFN‐γ control groups

During persistent *C*. *trachomatis* infection, uncovering the links between core functions or pathways with these differentially expressed mRNAs will further help us obtain deep insight into the key roles of these genes. The differentially expressed mRNAs showed an obvious trend of amplification at 24 hpi compared with 12 hpi; therefore, the 24 hpi chip data were analysed by GO and KEGG analysis. GO analysis was performed by biological process (BP), cellular components (CC) and molecular function (MF) and was composed of three parts. We found that the upregulated differential mRNAs were mainly enriched in biological processes related to nucleic acid metabolic process and RNA metabolic process, cellular components related to nuclear part and nuclear lumen, and molecular functions related to RNA binding and protein binding (Figure 3A, C, E). However, the downregulated differentially expressed mRNAs were mainly concentrated in biological processes related to cell fate specification and inclusion body assembly, cellular components related to intraciliary transport particle B and intrinsic components of the membrane, and molecular functions related to RAGE receptor binding and NAD binding (Figure 3B, D, F).

Pathway analysis of 1741 differentially expressed mRNAs was performed using KEGG analysis. In the persistent infection group, the upregulated differential mRNAs were mostly enriched in the NOD‐like receptor signalling pathway, herpes simplex infection, the TNF signalling pathway and the RIG‐I‐like receptor signalling pathway (Figure 3G). In addition, the downregulated differential mRNAs were mostly enriched in the TGF‐β, calcium and Hippo signalling pathways (Figure 3H). These results suggested that *C*. *trachomatis* might evade host immunity by affecting host signal transduction in persistent infection.

### 3.4 Validation of differentially expressed lncRNAs by quantitative real‐time PCR

**FIGURE 4:**
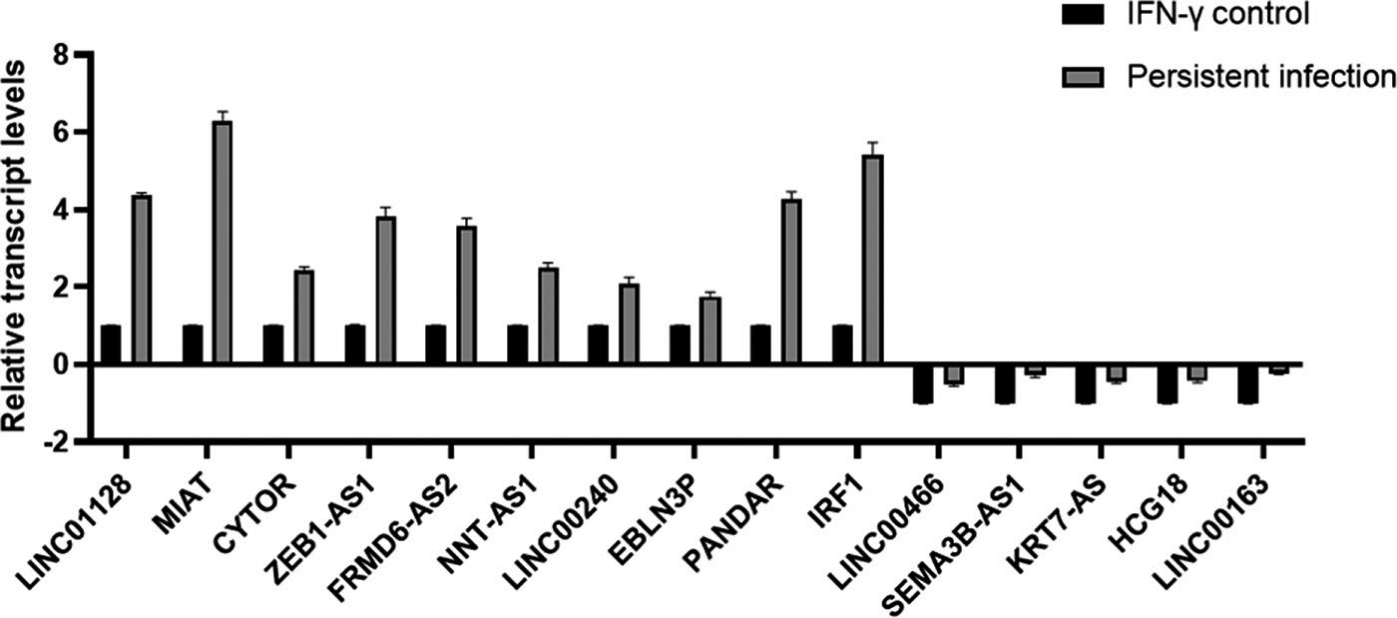
Validation of the microarray data using qRT–PCR. All data are representative of three independent experiments

The differential expression levels of lncRNAs screened in our microarray analysis were validated by qRT–PCR. We randomly selected 10 upregulated (LINC01128, MIAT, CYTOR, ZEB1‐AS1, FRMD6‐AS2, NNT‐AS1, LINC00240, EBLN3P, PANDAR, IRF1) and 5 downregulated (LINC00466, SEMA3B‐AS1, KRT7‐AS, HCG18, LINC00163) lncRNAs for their expression validation in the persistent infection group, and all were normalized by internal control *18S rRNA* expression. The results were similar to the trends observed in microarray data (Figure 4; Figure 2E).

### 3.5 MIAT, ZEB1‐AS1 and IRF1 are involved in apoptosis resistance in persistent infection

**FIGURE 5:**
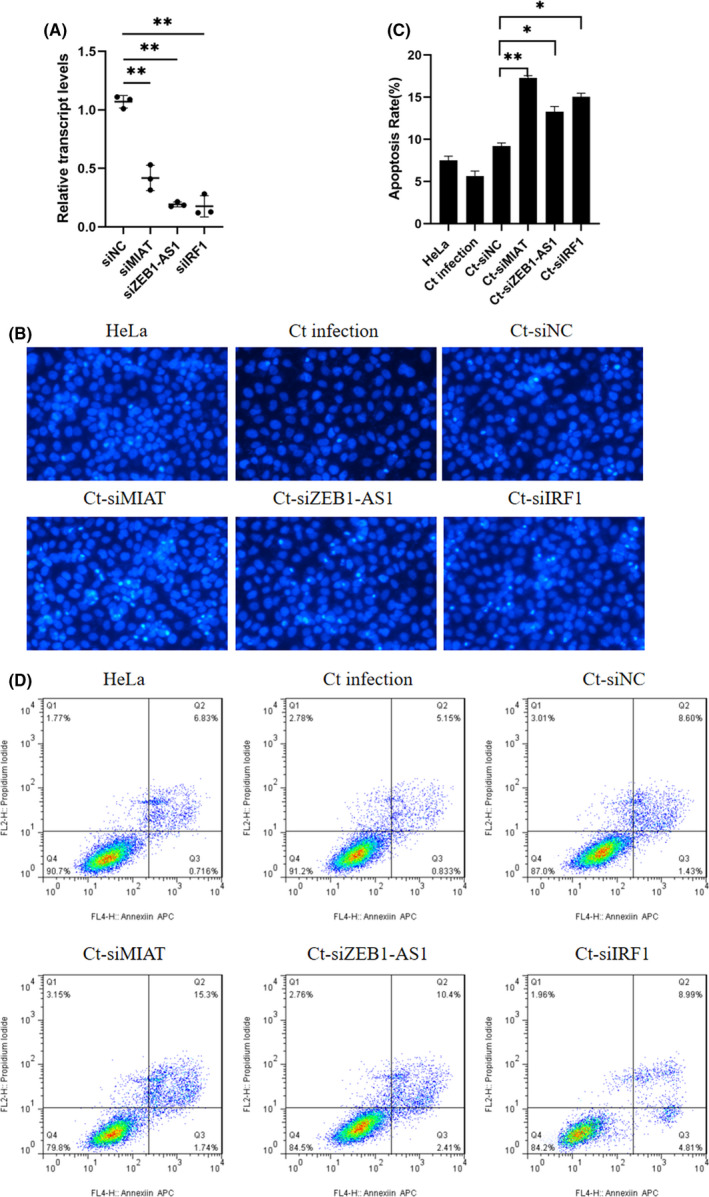
Key lncRNAs were involved in the antiapoptotic response induced by persistent *C*. *trachomatis* infection. (A) qRT–PCR was used to detect the effects of MIAT, ZEB1‐AS1 and IRF1 interference by siRNA. *18S rRNA* was used as an internal control, **p < 0.01. (B) The apoptosis rate was calculated by Hoechst staining (200×). HeLa cells were treated with siRNA for 24 h and then were subjected to persistent infection. (C) The apoptosis rate was calculated by Hoechst staining, *p < 0.05, **p < 0.01. (D) The apoptosis rate was calculated by FCM

*Chlamydia* can proliferate itself by resisting host cell apoptosis, which is one of the self‐protection mechanisms of *Chlamydia*.^21^, ^22^ However, many of the underlying molecular mechanisms responsible for host manipulation are still poorly understood. To investigate whether lncRNAs played a role in the antiapoptotic process of persistent *C*. *trachomatis* infection, LncATLAS and LNCipedia were performed to screen the differential lncRNAs in persistent infection, and the differentially regulated lncRNAs interacting with MIAT, ZEB1‐AS1 and IRF1 were selected for preliminary analysis. Through chip screening, the three lncRNAs were all upregulated in persistent infection, and studies have confirmed that they are closely related to apoptosis and tumour proliferation^23^, ^24^, ^25^; thus, we discuss the role of MIAT, ZEB1‐AS1 and IRF1 in the processes of persistent infection and anti‐host cell apoptosis. First, the expression levels of MIAT, ZEB1‐AS1 and IRF1 were inhibited by specific siRNAs and were significantly downregulated compared with siNC (*p* < 0.01) (Figure 5A). Then, Hoechst staining was used to detect the number of apoptotic bodies to determine the antiapoptotic status of each group. The results showed that the apoptosis rates of the Ct‐siMIAT, Ct‐siZEB1‐AS1 and Ct‐siIRF1 groups were 17.29% (*p* < 0.01), 13.27% (*p* < 0.05) and 15.08% (*p* < 0.05), respectively, which were higher than the 9.21% of the Ct‐siNC control group (Figure 5B, C). Subsequently, flow cytometry further verified the apoptotic effect of the Ct‐siMIAT, Ct‐siZEB1‐AS1 and Ct‐siIRF1 groups, and the results were essentially consistent with the Hoechst staining results (Figure 5D). In view of the previous GO and KEGG analysis results of differentially expressed mRNAs, we further explored the mechanism of the antiapoptotic function of MIAT, ZEB1‐AS1 and IRF1.

### 3.6 LncRNA‐miRNA‐mRNA interaction network analysis

**FIGURE 6:**
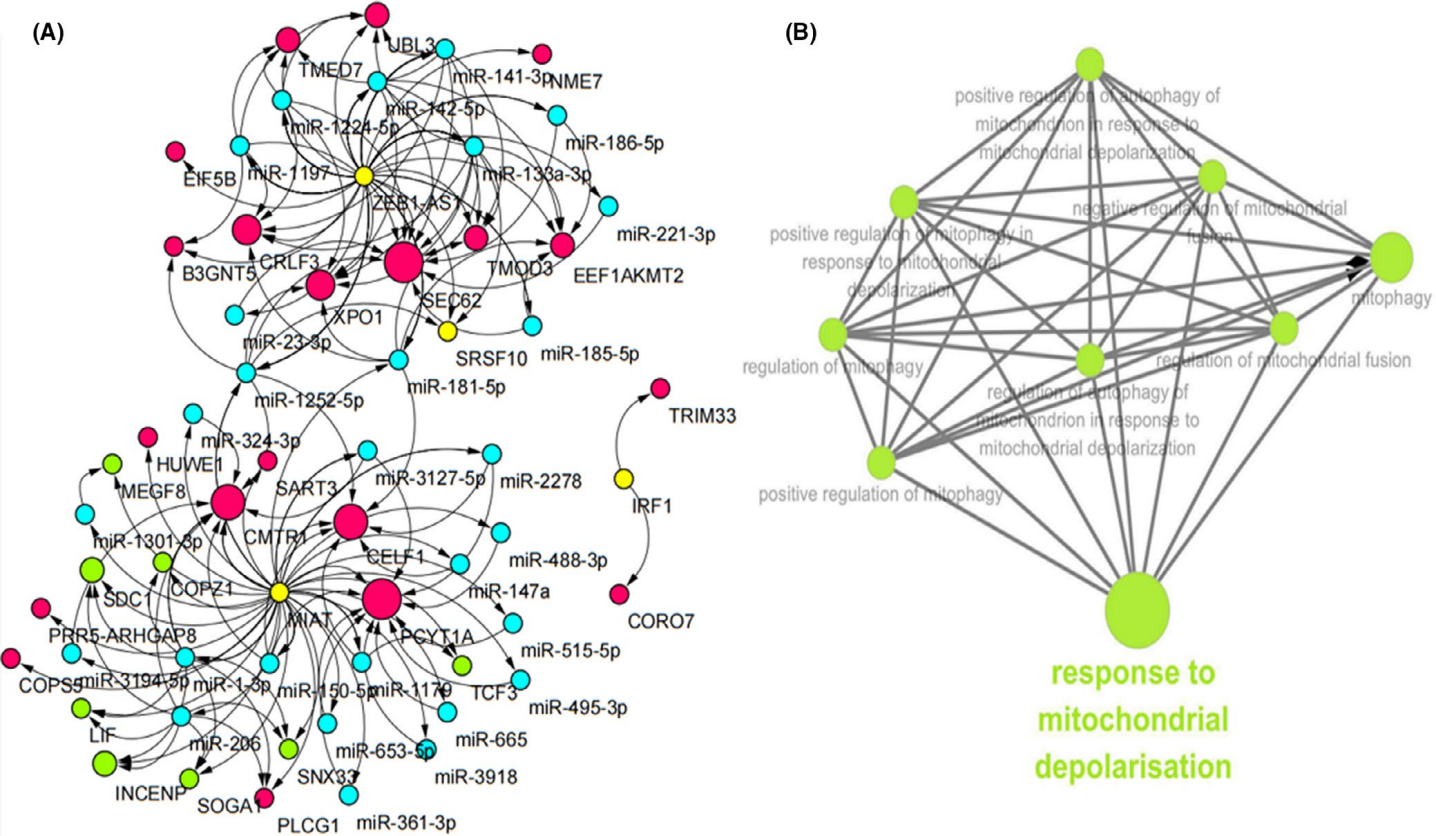
Construction and analysis of the lncRNA‐miRNA‐mRNA interaction network. (A) The lncRNA‐miRNA‐mRNA interaction network of key lncRNAs (MIAT, ZEB1‐AS1, IRF1). Yellow indicates lncRNAs, blue indicates microRNAs, red indicates upregulated mRNAs and green indicates downregulated mRNAs. The curved arrows represent targeted interacting relationships. (B) GO enrichment of MIAT‐targeted mRNAs. The green dots represent related biological processes

The ceRNA regulatory mechanism is very important between lncRNAs and mRNAs. Therefore, the analysis of specific RNA‐RNA interactions has become the key to exploring the mechanism of interaction between *Chlamydia* and the host. We predicted miRNAs and mRNAs of three lncRNAs (MIAT, ZEB1‐AS1 and IRF1) based on ENCORI and compared these mRNAs with the differentially expressed mRNAs that were identified in our chip results. To make the network more concise and effective, we further filtered the key mRNAs closely related with cell fate and the interferon response pathway (Figure 3G, H). A total of 29 mRNAs were selected for further lncRNA‐miRNA‐mRNA network construction (Figure 6A). These ceRNA regulatory relationships may be more important in the pathogenesis of persistent *C*. *trachomatis* infection considering the complexity amongst the lncRNAs and mRNAs. Furthermore, the ClueGo Plugin was used to analyse the targeted mRNAs of the three lncRNAs. Interestingly, we found that MIAT was involved in the response to mitochondrial depolarization (Figure 6B). Therefore, the following work will focus on the specific mechanism of MIAT.

### 3.7 The antiapoptotic effects of MIAT are related to the mitochondrial pathway

**FIGURE 7:**
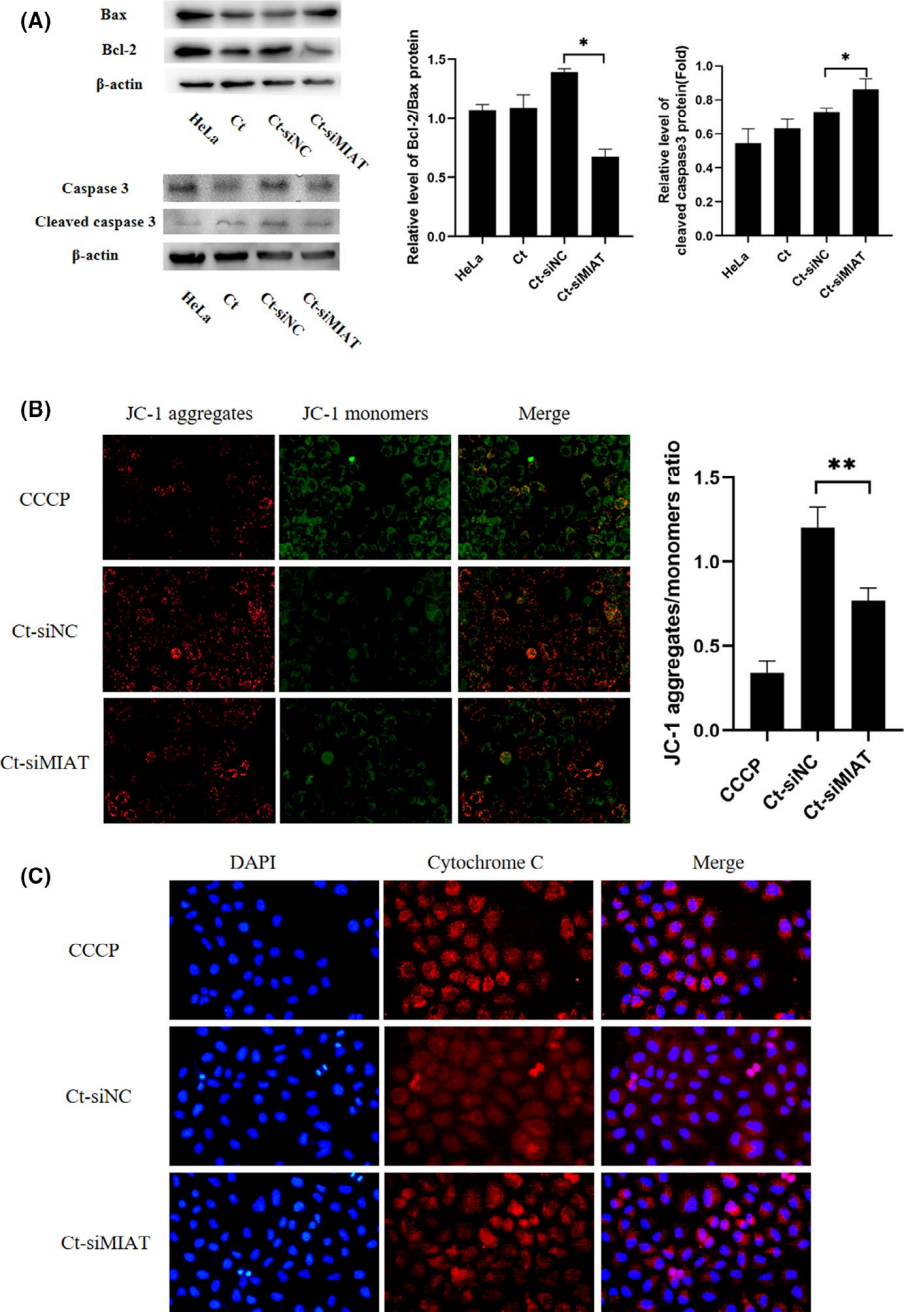
MIAT affects mitochondrial function during persistent *C*. *trachomatis* infection. (A) The indicated protein levels of Bax, Bcl‐2 and cleaved caspase‐3 were analysed by western blot. HeLa cells were treated with siMIAT for 24 h and then were subjected to persistent infection, *p < 0.05. (B) The fluorescence intensity of JC‐1 in mitochondria was observed by fluorescence microscopy (400×), and the positive control group was treated with 10 μm CCCP for 20 min before detection. (C) Cytochrome c fluorescence intensity per cell was assayed by fluorescence microscopy (400×). HeLa cells were treated with siMIAT for 24 h and then persistently infected, **p < 0.01

In recent years, many studies have shown that the mitochondrial pathway in the cell survival mechanism plays a vital role,^26^, ^27^ and in numerous apoptosis control genes, the Bcl‐2 and caspase families are of the most concern.^28^, ^29^ Amongst them, the Bcl‐2 and Bax genes are known to regulate the apoptosis regulation function of a counterpart pair of the most important control genes. Caspase‐3 is the most critical apoptotic executive protease during the process of apoptosis.^30^, ^31^, ^32^ To further understand the antiapoptotic mechanism of MIAT in persistent infection, we tested the classical apoptosis‐related molecules Bax, Bcl‐2 and cleaved caspase‐3 in the Ct‐siMIAT group. Compared with the Ct‐siNC group, the Bcl‐2/Bax ratio of the Ct‐siMIAT group was downregulated, and the ratio of cleaved caspase‐3 to the internal reference was increased (*p* < 0.05) (Figure 7A). Decreased MMP is one of the important factors leading to apoptosis and is considered to be the first step of the apoptosis cascade.^33^ To further investigate whether MIAT affected mitochondrial permeability by changing MMP, a JC‐1 fluorescent probe was used to detect MMP, and carbonyl cyanide 3‐chlorophenylhydrazone (CCCP) was used as a positive control to induce MMP decline. In most cells, MMP is reduced after CCCP treatment, and JC‐1 staining shows green fluorescence, whilst normal cells should show red fluorescence. Our results showed that the MMP of the Ct‐siMIAT group was significantly lower than that in the Ct‐siNC group, and the ratio of JC‐1 aggregates/monomers decreased approximately 2‐fold if MIAT was silenced (*p* < 0.01) (Figure 7B). In addition, considering that the change in mitochondrial permeability could lead to the release of cytochrome c to promote cell apoptosis, we detected the release of cytochrome c by immunofluorescence, and the results showed that the content of cytochrome c in the cytoplasm of the Ct‐siMIAT group was significantly increased (Figure 7C). These results confirmed that MIAT inhibited the release of cytochrome c from the mitochondria into the cytosol by attenuating mitochondrial membrane permeability. Thus, MIAT‐regulated mitochondria‐mediated host cell apoptosis in persistent *C*. *trachomatis* infection.

### 3.8 Inhibition of MIAT promotes *C. trachomatis* growth and development

**FIGURE 8:**
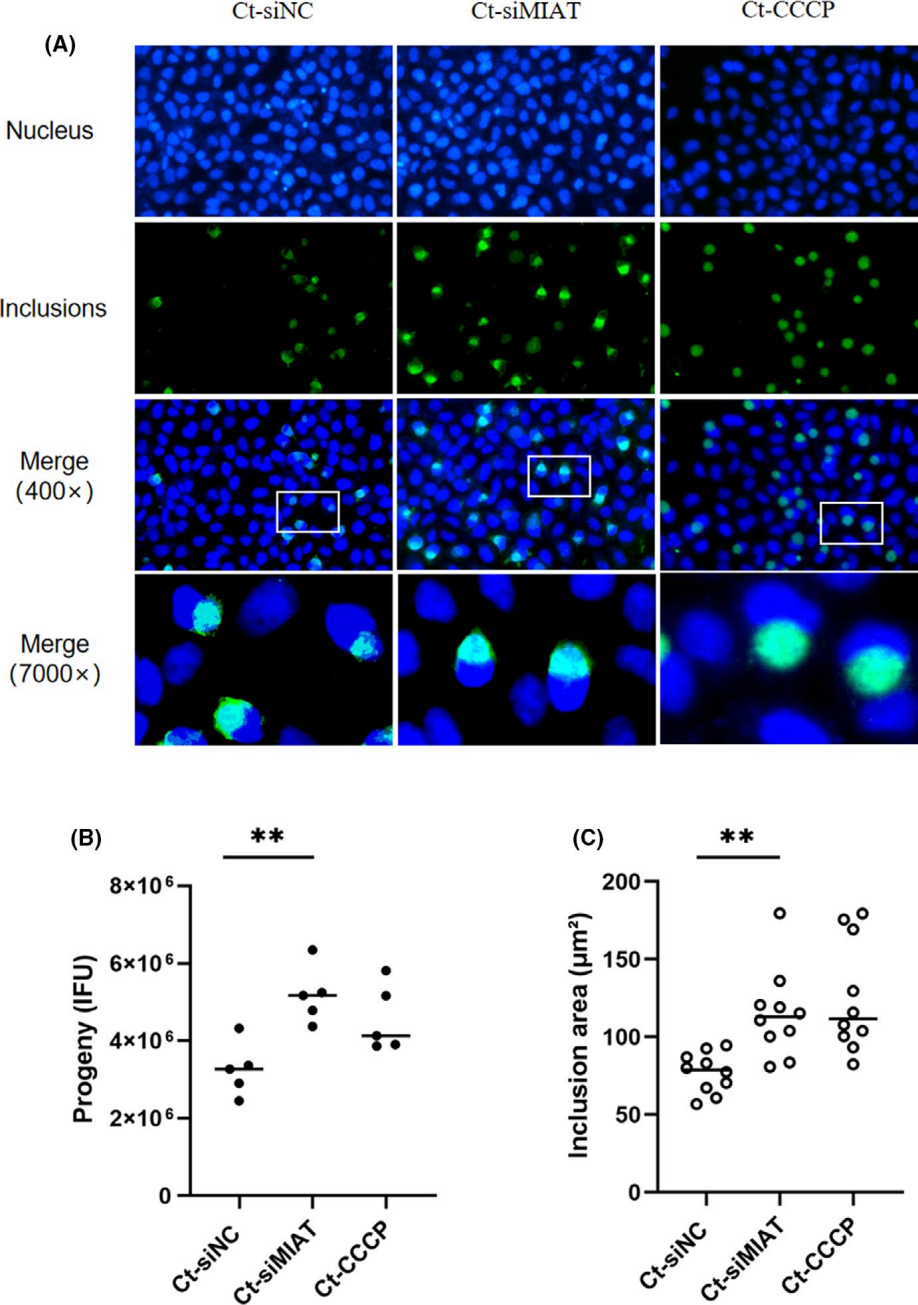
LncRNA MIAT was involved in the development of *C*. *trachomatis* in persistent infection. (A) Effect of siMIAT on the growth and development of *C*. *trachomatis* (400×). HeLa cells were treated with siMIAT for 24 h and then persistently infected. The positive control group was treated with 10 μm CCCP for 20 min. DNA (blue) was stained with Hoechst 33258, and inclusions (green) were stained with Cy2. (B) The progeny of groups were harvested for counting (IFUs). Progeny were harvested at 40 hpi. **p < 0.01. (C) Morphological changes of inclusions (7000×). The areas were counted by ImageJ. **p < 0.01

During persistent *Chlamydia* infection, in addition to changes in morphology, the expression profiles of genes involved in cell biological processes such as membrane structure, energy metabolism and development cycle regulation also show different degrees of change.^34^, ^35^, ^36^ Abnormal bodies showed decreased metabolic activity and replication ability in cells. German researchers studied the regulation of mitochondrial fission regulators by host miRNAs, which, in turn, severely affected chlamydial growth and had a marked effect on the mitochondrial network.^37^ A reduced mitochondrial membrane potential has been shown to correlate with the reduced antiviral response.^38^ Because the functional lncRNA MIAT can cause decreases in the host cell mitochondrial membrane potential, we hypothesized that MIAT may influence chlamydial development. Therefore, we examined the effect of MIAT on *C*. *trachomatis* development. When MIAT was silenced, we found that the size of the *Chlamydia* inclusions was larger at 24 hpi. To investigate whether the decrease in mitochondrial membrane potential is conducive to the transformation of RB to EB, thus affecting the development of *Chlamydia*, we treated CCCP for 20 min as the positive control and then collected the progeny *Chlamydia* for reinfection counts (Figure 8A). Each group was given an identical volume to infect fresh HeLa cell monolayers. Compared with the Ct‐siNC group, the Ct‐siMIAT group progeny had (5.18 ± 0.25) × 10^6^ IFUs, which was higher than that of the Ct‐siNC group, at (3.26 ± 0.35) × 10^6^ IFUs. The IFUs of *C*. *trachomatis* were significantly increased (*p* < 0.01) (Figure 8B). The average diameter of the inclusions in the Ct‐siMIAT group was 1.5‐fold that of the negative control group (*p* < 0.01) (Figure 8C). Except for their larger size, the inclusion bodies had a normal appearance. The above results indicated that MIAT was involved in the host regulation of the growth and development of *Chlamydia* during persistent infection.

## 4 DISCUSSION

Persistent *C*. *trachomatis* infection is considered a survival strategy to evade host immune effects in vitro and in vivo.^39^, ^40^, ^41^ Most previous studies have been based on the interaction between bacterial virulence proteins and host coding genes.^42^, ^43^, ^44^ However, interestingly, 98.5% of the human genome is noncoding, and lncRNAs have emerged as important regulators of gene expression. A large amount of evidence has shown that lncRNAs are involved in the regulation of apoptosis pathways.^45^, ^46^ However, apoptosis is an important host defence mechanism against pathogens, and the regulation of *C*. *trachomatis* infection on apoptosis is quite complex.^47^, ^48^, ^49^ Currently, it is believed that *C*. *trachomatis* can proliferate itself by resisting host cell apoptosis, and some studies have also shown that the enlargement of *Chlamydia* inclusions would promote cell apoptosis.^50^

In this study, we combined our previous work^51^, ^52^ to propose the scientific hypothesis that persistent *C*. *trachomatis* infection‐related differential lncRNAs might be involved in host cell antiapoptotic activity. Subsequently, we used a microarray platform to perform transcriptome analysis of lncRNAs and protein‐coding genes in an in‐vitro cell model of persistent *C*. *trachomatis* infection. Through GO, KEGG and lncRNA‐miRNA‐mRNA interaction network analyses, we screened out the key functional molecules from a large number of differentially expressed lncRNAs. The bioinformatics analysis of the screened lncRNAs revealed that IRF1 targets were enriched in posttranslational protein targeting to the membrane and translocation, whilst MIAT targets were enriched in response to mitochondrial depolarization, and ZEB1‐AS1 targets were mostly related to mRNA splice site selection and spliceosomal tri‐snRNP complex assembly.^53^ These functional analyses provided a reference for us to explore the different specific mechanisms involved.

LncRNAs are involved in the regulation of host cell apoptosis during pathogen infection, which has attracted extensive attention.^54^, ^55^ For instance, HBV‐upregulated SAMD12‐AS1 regulated cell proliferation and apoptosis via the NPM1‐HDM2‐p53 axis.^56^ In BCG‐infected macrophages, the downregulation of LincRNA‐EPS inhibited apoptosis by activating the JNK/MAPK signalling pathway.^57^ In enterovirus‐71 infection, lnc‐IRAK3‐3 has the ability to capture miR‐891b to enforce GADD45β expression and eventually promote apoptosis.^58^ In persistent *C*. *trachomatis* infection, MIAT, ZEB1‐AS1 and IRF1 were involved in apoptosis resistance, as determined by Hoechst staining and flow cytometry. Remarkably, in our work, we used specific siRNA for functional verification. Despite the initial results showing excellent specificity in siRNA‐mediated gene silencing, many studies have shown that siRNA can also produce multiple specific and nonspecific mechanisms other than the intended lncRNA inhibition. In addition, compared with the acute infection data, only 196 differentially expressed lncRNAs were found in persistent infection, most of which had the same differential expression (Figure S1 and Table S4). From these chip data, we determined that a large number of differentially expressed lncRNAs were induced by *C*. *trachomatis* and that the pORF5 plasmid protein is an important virulence factor for *C*. *trachomatis*. Based on these findings, we believe that the lncRNA profile of the pORF5 deletion strain can explain whether the antiapoptotic activity of lncRNA induced by *C*. *trachomatis* requires the involvement of pORF5. This will help us further study the key molecules responsible for *C*. *trachomatis* infection.

Numerous studies have shown that lncRNAs affect pathogen survival by regulating cell metabolism and innate immunity. Subuddhi et al. found that the lncRNA HOTAIR can affect the production of reactive oxygen species (ROS) during infection by regulating the expression of SATB1 and DUSP4, thus promoting the survival of virulent *M*. *tuberculosis*.^59^ Hongwei et al. found that lncRNA NEAT1 promoted interferon responses by acting as positive feedback for RIG‐I signalling and then controlled Hantaan virus replication.^60^ In adapting to intracellular life, *Chlamydia* has lost energy‐generating capability but has modified nucleotide transport functions to exploit the host ATP.^61^ In this study, we found that MIAT can influence MMP, thereby regulating the growth and development of *C*. *trachomatis* during persistent infection. Infection‐induced changes in mitochondrial homeostasis were associated with increases in mitochondrial respiratory activity, ATP production, and intracellular growth in *C*. *trachomatis*. Our observations raise the possibility of harnessing lncRNAs to develop host‐directed therapies for persistent *C*. *trachomatis* infection.

Notably, lncRNAs act in different mechanisms based on their cellular location. Numerous studies have shown that different subcellular localizations of lncRNAs determine their regulation mode.^62^, ^63^ It is generally believed that lncRNA localization in the nucleus is transcriptional regulation and plays a role in the nucleus by internuclear factors.^64^ Through the lncATLAS database, we found that MIAT accounted for 99.1% of nuclear localization; thus, we speculated that it might play an important role in the regulation of chromatin, transcriptional regulation and variable shear regulation. LncRNAs located in the cytoplasm may regulate gene expression at the posttranscriptional level through the ceRNA mechanism^65^; for example, ZEB1‐AS1 can affect the expression of host mRNA through direct binding of mRNA or competitive binding of miRNA.^66^ According to the analysis, the majority of IRF1 was located in the cytoplasm and exosomes. Therefore, the study of its cellular sublocalization becomes a key step to explore the mechanism in depth. The regulation of lncRNAs in persistent *C*. *trachomatis* infection is a synergistic effect of multiple mechanisms. Our study implies that MIAT is not only a previously unknown molecule for *C*. *trachomatis* growth suppression following persistent infection but also a potential target for future therapy against *C*. *trachomatis*‐mediated chronic inflammation.

## CONFLICT OF INTEREST

The authors declare that they have no conflicts of interest with the contents of this article.

## AUTHOR CONTRIBUTION

**Fangzhen Luo:** Conceptualization (equal); Data curation (equal); Formal analysis (equal); Validation (equal); Visualization (equal); Writing – original draft (equal). **Yating Wen:** Conceptualization (equal); Validation (equal). **Lanhua Zhao:** Data curation (supporting); Supervision (supporting). **Shengmei Su:** Conceptualization (supporting); Software (supporting). **Yuqi Zhao:** Investigation (supporting). **Wenbo Lei:** Conceptualization (supporting). **Zhongyu Li:** Conceptualization (equal); Methodology (lead); Project administration (lead); Resources (lead); Supervision (lead).

## Supporting information

Figure S1

Tables S1–S4

## Data Availability

The data that support the findings of this study are available from the corresponding author upon request.
