# Supplementary figures and images for: Chlamydia trachomatis induces lncRNA MIAT upregulation to regulate mitochondria‐mediated host cell apoptosis and chlamydial development
Source: J Cell Mol Med. 2021 Dec 3;26(1):163–77. doi: 10.1111/jcmm.17069 (PMC8742237; doi:10.1111/jcmm.17069)

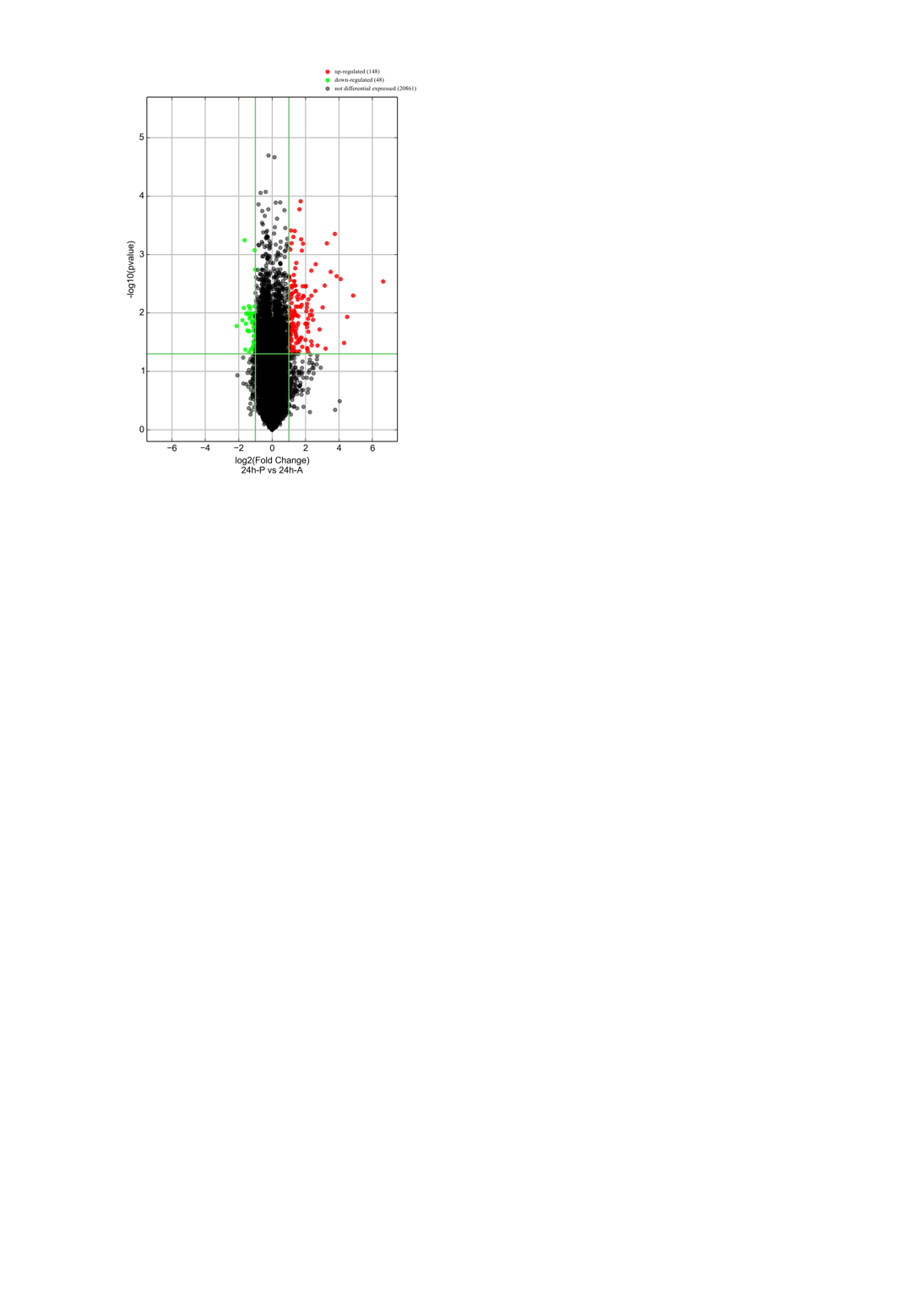

Supplement: Supplementary file 1 — Figure S1 [file JCMM-26-163-s001.tif]
