# Supplementary material for: Chlamydia trachomatis induces lncRNA MIAT upregulation to regulate mitochondria‐mediated host cell apoptosis and chlamydial development
Source: J Cell Mol Med. 2021 Dec 3;26(1):163–77. doi: 10.1111/jcmm.17069 (PMC8742237; doi:10.1111/jcmm.17069)
Supplement: Supplementary file 2 — Tables S1–S4 [file JCMM-26-163-s002.doc]

| **Supplementary Table 1. Primers designed for qRT-PCR validation of candidate lncRNAs** | | | | | | |
| --- | --- | --- | --- | --- | --- | --- |
| GeneSymbol | Forward | | | | Reverse | |
| MIAT | TTGCAGGAGAGAGAAGTGGG | | | | ACTGGAGGTGAGGCATGAAA | |
| CYTOR | GACATTCCAGACAAGCGGTG | | | | TGGTTCCCGTTATCAGGTCC | |
| ZEB1-AS1 | TGCTTGTCTCACTTCCCCAT | | | | AGGAATTCATGGCCTGTGGA | |
| FRMD6-AS2 | GTACACTCAGAGGCCACACT | | | | GTTAGGTCCTAGTGGCAGCA | |
| NNT-AS1 | CCTCACGAAATGCCCCTTTG | | | | GGGGAGAAGCAAATGTGTCC | |
| LINC00240 | CAACCTCTCCTCTGGATGCT | | | | CATCCTCCTCTTCAGACCGG | |
| EBLN3P | CAGCAGTCTCCAGGTTCAGA | | | | GCTCTTTCCCTGGAGACTCA | |
| PANDAR | CCAGCTGGCAATCTACAACC | | | | CTGAGGGGAGGCTCATACTG | |
| IRF1 | GCCTTCTTCCCTCTTCCACT | | | | CCTCGATATCTGGCAGGGAG | |
| LINC00163 | GAAAGTGCAGCAGAGACCTC | | | | TTGCTATTTTCTCCACGCGG | |
| LINC00466 | TGTTACGTCCAGGGTCTCAC | | | | TCATTCTAGGCTGGGTGTGG | |
| SEMA3B-AS1 | CTCAACCTCTCCCTCCACAG | | | | ACCAGACTCAGCTTCAGGAC | |
| KRT7-AS | CTTGGAGCAGGAATCAGCAC | | | | TGTGCGGCTTCTTAGGGTTA | |
| HCG18 | GATGTGGTCAGGAGTGGGAA | | | | TAAGTTACCATGGCCCAGCA | |
| LINC01128 | CAGTACGTACCCCAGTGTGT | | | | GATCACTCCAGCCTCATCCA | |
| *18S rRNA* | CGCTCGCTCCTCTCCTACTT | | | | CGGGTTGGTTTTGATCTGATAA | |
| **Supplementary Table 2. Top 20 deregulated lncRNAs detected using microarray** | | | | | | |
| Up-regulated lncRNAs | | Fold change |  | Down-regulated lncRNAs | | Fold change |
| AC116366.3 | | 86.9928033 |  | HSALNG0016500 | | 25.7085137 |
| LINC01619 | | 83.3243903 |  | AC092902.2 | | 18.0635263 |
| MAN1B1-DT | | 83.3243903 |  | RCAN1 | | 16.5062038 |
| G048845 | | 83.3243903 |  | XLOC_005435 | | 14.4551262 |
| AC011484.1 | | 83.3243903 |  | G079839 | | 10.8635638 |
| ZBED5-AS1 | | 83.3243903 |  | G075807 | | 10.4887217 |
| AC020915.3 | | 35.0383662 |  | MLF1 | | 9.2861945 |
| HIF1A-AS1 | | 29.649852 |  | AC093817.1 | | 7.6223693 |
| NAMA | | 21.7302821 |  | G022638 | | 7.6223693 |
| G033488 | | 21.7302821 |  | XLOC_002727 | | 7.5527315 |
| AF117829.1 | | 21.7302821 |  | LINC01992 | | 7.5527315 |
| TXLNGY | | 21.7302821 |  | AL354928.1 | | 7.5527315 |
| AL139260.2 | | 21.7302821 |  | G079765 | | 7.30772 |
| CIP2A | | 15.9031043 |  | LINC00839 | | 7.2085515 |
| TXNDC12 | | 15.6430221 |  | XLOC_011649 | | 7.1912624 |
| G064362 | | 14.8312881 |  | AC113146.1 | | 7.1912624 |
| NEDD4L | | 11.9696964 |  | G035763 | | 6.9147982 |
| C9orf62 | | 11.3560209 |  | CATG00000043895.1 | | 6.9147982 |
| AC087481.3 | | 11.1796696 |  | AC110285.1 | | 6.9147982 |
| MANEA-DT | | 11.0007311 |  | AC144831.1 | | 6.9147982 |

| **Supplementary Table 3. Top 20 deregulated mRNAs detected using microarray** | | | | | | |
| --- | --- | --- | --- | --- | --- | --- |
| Up-regulated mRNAs | Genes | Fold change |  | Down-regulated mRNAs | Genes | Fold change |
| NM_153269 | C20orf96 | 1039.670496 |  | NM_201589 | MAFA | 52.7932856 |
| NM_001080485 | ZNF275 | 917.5649037 |  | NM_144999 | LRRC45 | 17.9251247 |
| NM_001294145 | SSB | 745.8503076 |  | NM_003280 | TNNC1 | 13.068923 |
| NM_001204803 | ANO6 | 455.7575356 |  | NM_001288957 | IFI27 | 12.9164271 |
| NM_001130475 | THAP5 | 448.3447363 |  | NM_001270639 | JOSD2 | 11.1445252 |
| NM_001004705 | OR4D10 | 185.1925882 |  | NM_001172702 | SLC38A6 | 9.7142048 |
| NM_015063 | SLC8A2 | 181.23688 |  | NM_016524 | SYT17 | 9.3191492 |
| NM_007062 | PWP1 | 136.605518 |  | NM_012344 | NTSR2 | 8.8428674 |
| NM_138798 | MITD1 | 77.318093 |  | NM_178545 | TMEM52 | 6.9042227 |
| NM_032288 | FYTTD1 | 64.596966 |  | NM_001130448 | C15orf62 | 6.7011409 |
| NM_016289 | CAB39 | 49.3001148 |  | NM_016429 | COPZ2 | 6.1892271 |
| NM_001244753 | FCGR3B | 47.2329176 |  | NM_031941 | USHBP1 | 5.9702169 |
| NM_001286274 | KIAA1586 | 43.0928452 |  | NM_005346_5 | HSPA1B | 5.5551361 |
| NM_007172 | NUP50 | 42.9171271 |  | NM_001136108 | R3HCC1 | 5.368367 |
| NM_001145547 | RBM17 | 42.1845074 |  | NM_002705 | PPL | 5.2666223 |
| NM_001145418 | TTC28 | 33.8351763 |  | NM_002167 | ID3 | 5.2446558 |
| NM_003059 | SLC22A4 | 31.861083 |  | NM_054031 | MRGPRX3 | 5.1382444 |
| NM_032117 | MND1 | 31.8158079 |  | NM_001024807 | APLP1 | 5.092447 |
| NM_016436 | PHF20 | 30.7770703 |  | NM_001159 | AOX1 | 5.0900995 |
| NM_001040664 | PPAN-P2RY11 | 28.4234821 |  | NM_024111 | CHAC1 | 5.0272589 |

| **Supplementary Table 4. Top 20** **deregulated lncRNAs detected using microarray (24h-persistent infection_vs_24h-acute infection)** | | | | |
| --- | --- | --- | --- | --- |
| Up-regulated lncRNAs | Fold change |  | Down-regulated lncRNAs | Fold change |
| SMAD1 | 99.9119163 |  | HSALNG0016500 | 4.367103 |
| XLOC_005173 | 22.4387367 |  | AL391839.1 | 4.2256022 |
| LINC01619 | 22.4387367 |  | RCAN1 | 2.9308142 |
| MAN1B1-DT | 22.4387367 |  | LINC02593 | 2.9180944 |
| RGS5 | 22.4387367 |  | AL162413.1 | 2.9180944 |
| AL512328.1 | 17.1457368 |  | AC109466.1 | 2.7883874 |
| GIGYF2 | 17.1457368 |  | FOXP4-AS1 | 2.7697547 |
| PPP4R1-AS1 | 13.4613286 |  | G068616 | 2.7219494 |
| PCAT7 | 9.6791583 |  | LINC00378 | 2.6866092 |
| MIR7515HG | 9.6791583 |  | SNHG15 | 2.6866092 |
| AC093534.2 | 9.6791583 |  | XLOC_006312 | 2.6866092 |
| OBSCN | 9.6791583 |  | CATG00000088098.1 | 2.6866092 |
| AC009055.1 | 8.8492734 |  | AL121983.2 | 2.674119 |
| G048845 | 8.8492734 |  | AC083798.2 | 2.674119 |
| AL035563.1 | 8.8492734 |  | AC159540.1 | 2.674119 |
| XLOC_013420 | 8.8492734 |  | AP001496.4 | 2.674119 |
| AL109933.1 | 8.8492734 |  | LINC01366 | 2.674119 |
| LINC01104 | 8.8492734 |  | ART3 | 2.674119 |
| XLOC_005435 | 8.1201428 |  | G051320 | 2.5473927 |
| THAP9-AS1 | 8.1201428 |  | AL596094.1 | 2.509927 |
